# UVC-Induced Oxidative Stress and DNA Damage Repair Status in Head and Neck Squamous Cell Carcinoma Patients with Different Responses to Nivolumab Therapy

**DOI:** 10.3390/biology14020195

**Published:** 2025-02-13

**Authors:** Christina Papanikolaou, Panagiota Economopoulou, Niki Gavrielatou, Dimitra Mavroeidi, Amanda Psyrri, Vassilis L. Souliotis

**Affiliations:** 1 Institute of Chemical Biology, National Hellenic Research Foundation, 11635 Athens, Greece; chrpapa@eie.gr (C.P.); dmavro@eie.gr (D.M.); 2 Second Department of Internal Medicine, Medical Oncology Section, National and Kapodistrian University of Athens, Attikon University Hospital, 12462 Athens, Greece; panagiota_oiko@hotmail.com (P.E.); nikigavrielatou@gmail.com (N.G.); psyrri237@yahoo.com (A.P.)

**Keywords:** head and neck squamous cell carcinoma, DNA damage repair, oxidative stress status, immune checkpoint blockade, apurinic/apyrimidinic lesions, endogenous/baseline DNA damage

## Abstract

Accumulation of evidence highlighted the crosstalk between DNA damage repair and the immune system. Herein, we tested the hypothesis that in head and neck squamous cell carcinoma (HNSCC), the DNA repair capacity of patients’ PBMCs correlates with therapeutic response to immune checkpoint blockade. Following in vitro UVC irradiation, oxidative stress, apurinic/apyrimidinic (AP) lesions, endogenous/baseline DNA damage, and DNA damage repair efficiency were evaluated in three HNSCC (UM-SCC-11A, Cal-33, BB49) and two normal cell lines (RPMI-1788, 1BR-3h-T), as well as in peripheral blood mononuclear cells (PBMCs) from 15 healthy controls (HC) and 49 recurrent/metastatic HNSCC patients at baseline (8 responders, 41 non-responders to subsequent nivolumab therapy). HNSCC cell lines showed lower DNA repair efficiency, increased oxidative stress, and higher AP sites than normal ones (all *p* < 0.001). Moreover, patients’ PBMCs exhibited increased endogenous/baseline DNA damage, decreased DNA repair capacity, augmented oxidative stress, and higher AP sites than PBMCs from HC (all *p* < 0.001). Importantly, PBMCs from responders to nivolumab therapy showed lower endogenous/baseline DNA damage, higher DNA repair capacities, decreased oxidative stress, and reduced AP sites than non-responders (all *p* < 0.05). Together, we demonstrated that oxidative stress status and DNA repair efficiency in PBMCs from HNSCC patients are correlated with the response to immune checkpoint blockade.

## Simple Summary

Immune-directed therapy reactivates the body’s immune system to fight against cancer cells. In particular, immune checkpoint inhibitors (ICIs), a major class of immunotherapy drugs, have improved survival rates and provided long-lasting results for many cancer patients. However, response rates differ significantly both within and among different types of cancers. Therefore, significant challenges to increasing the response rates and to identifying sensitive and specific biomarkers for responses to ICI treatment remain. Recent data highlight that alterations in the DNA damage repair of the tumor cells can activate an immune response and seem to be associated with patient survival and immunotherapy response. This interesting interplay between the DNA damage repair status and the immune system has opened up new perspectives in clinical studies for cancer treatment. Herein, we found that alterations in the oxidative stress status and the DNA repair capacity of peripheral blood mononuclear cells (PBMCs) from head and neck squamous cell carcinoma (HNSCC) patients at baseline are implicated in the response to subsequent immune checkpoint blockade. If additional studies confirm these findings, they can lead to the development of new predictive biomarkers to immunotherapy in HNSCC and may drive the design of novel ICI-based combination therapies.

## 1. Introduction

Head and neck squamous cell carcinoma (HNSCC) comprises a diverse collection of tumors that occur in the oral cavity, pharynx, and larynx. These highly immune-infiltrated malignancies are defined by a tumor microenvironment (TME) that is primarily immunosuppressive [1]. Etiologically, HNSCC is correlated with alcohol consumption, tobacco use, and infections with the Epstein–Barr virus (EBV) and human papillomavirus (HPV) [2]. HNSCC is a malignancy with a severe impact on a patient’s quality of life, mostly due to the severe side effects of treatment [3]. HNSCC is usually treated with surgery, chemotherapy, and radiotherapy [4]. Nowadays, immune checkpoint inhibitors (ICIs) have become the revolutionized standard of treatment of recurrent/metastatic disease as monotherapy or in combination with chemotherapeutics. Thus, translational research is now focused on the content and pathophysiology of TMEs to fully characterize the unique components and interactions that impact anti-tumor immunity and to discover new predictive biomarkers for immunotherapy [5].

Daily, our cells accumulate DNA lesions that have the potential to impede fundamental cellular functions, including transcription and genome replication. If these lesions are not correctly repaired, they may also lead to mutations or aberrations in the genome, which are associated with human diseases, including cancer [6]. These DNA lesions are the result of several exogenous causes, including ultraviolet (UV) and ionizing radiation, various chemical agents, as well as endogenous factors, such as mismatching of DNA bases, DNA alkylation, hydrolysis, and oxidation [7]. Cells have created a network of mechanisms, known as DNA damage response (DDR), to detect and repair damages in response to these DNA changes, safeguarding the integrity of the genome [8,9]. DDR is a well-organized system comprising sensors, mediators, transducers, and effectors that activate several pathways, including cell cycle and DNA repair. Apoptosis or mutagenesis is triggered if the amount of unrepaired DNA lesions exceeds a predetermined threshold [10,11]. Specifically, the detection of a DNA lesion activates DDR. A cell signaling pathway is triggered, which leads to the induction of complex mechanisms for genome protection (apoptosis, cell cycle checkpoints, DNA repair pathways, transcription, and chromatin remodeling). Deregulated DDR, on the contrary, may cause mutagenesis and genomic instability [8]. As DDR controls the cellular choice of whether to eliminate DNA damage or initiate apoptosis, it plays a role in the pathogenesis and progression of multiple diseases, as well as their therapeutic response [12,13,14].

Importantly, recent studies have shown that alterations in the tumor-related DDR network affect both immune surveillance and immune responses, potentially augmenting the efficacy of immunotherapy [15,16]. There are several ways that a defective DDR network may boost the antitumor immune response. For example, increased tumor mutations and elevated level of cell surface neoantigens that T lymphocytes can identify, are the results of a deficiency in DNA damage repair [17]. In addition, DDR failure may result in the induction of cytosolic DNA. This DNA attaches to cGAS (cyclic guanosine monophosphate–adenosine monophosphate synthase) and then triggers the innate immune response through the STING (stimulator of interferon genes) pathway [18]. Furthermore, inhibition of the ataxia-telangiectasia mutated (ATM) kinase promotes an innate immune response mediated by interferons in a way that is dependent on the proto-oncogene tyrosine protein kinase SRC and the TANK-binding kinase 1 (TBK1) [19]. Particularly, research on HNSCC has demonstrated that the DNA damage caused by genotoxic drugs and the associated cellular reactions enhances tumor immunogenicity and the efficacy of immune checkpoint blockade therapy [20]. Collectively, these data suggest that immune checkpoint inhibitors may be more effective against cancers with underlying DNA repair abnormalities and that targeting DDR may be an advantageous approach to increasing the effectiveness of immune checkpoint inhibition [13].

In a similar vein, oxidative stress emerges when there is an imbalance between the generation of reactive oxygen species (ROS) and the capacity of cells to remove them. This imbalance can trigger proto-oncogenes and silence cancer suppressor genes [21]. Oxidative stress has the potential to cause oxidative damage to fundamental cellular constituents (proteins, lipids, and DNA), thus advancing the pathophysiology and progression of the disease. Patients with HNSCC, in particular, have been discovered to have a compromised antioxidant system and elevated oxidative stress [13,22,23]. Interestingly, oxidative stress influences the phenotype and function of myeloid DCs (dendritic cells) within the TME, and affects the functional behavior of tumor T regulatory cells (Tregs), which in turn diminishes the response to immune checkpoint inhibitors [24]. However, it is still unknown how precisely oxidative stress contributes to the onset and progression of HNSCC, as well as the response to therapy.

Here, we examined the hypothesis that oxidative stress and DNA repair efficiencies measured in peripheral blood mononuclear cells (PBMCs) from HNSCC patients correlate with the response to ICIs. We followed a systematic approach to evaluate several DDR parameters in normal and HNSCC cell lines, as well as in PBMCs from healthy controls and HNSCC patients with different response rates to nivolumab therapy.

## 2. Materials and Methods

### 2.1. Patients

Our study included 49 recurrent/metastatic (R/M) HNSCC patients (Table 1) who participated in a phase II nivolumab trial (NCT03652142; protocol #ΒΠΠΚ, ΕΒΔ 257/18-05-2020). As for the study design, in brief, tissue biopsies and peripheral blood were prospectively collected from 60 patients at baseline and, following safety and accessibility assessments, at two additional timepoints: after two cycles of nivolumab (240 mg intravenously every two weeks) and at disease progression. Imaging follow-ups were performed every three months during treatment. The best overall response to immunotherapy was annotated based on the response evaluation criteria in solid tumors (RECIST), version 1.1. Responses were categorized as complete response (CR), partial response (PR), stable disease (SD), or progressive disease (PD) [25]. Epidemiological and clinical data, as well as progression-free survival (PFS) and overall survival (OS), were also calculated from the database, with data as of 1 August 2021. A CONSORT diagram is shown in Figure S1. PBMCs were isolated from peripheral blood and purified according to established protocol [13]. PBMCs were resuspended in a cryopreservation medium consisting of 90% fetal bovine serum (FBS) and 10% dimethyl sulfoxide, then stored at −80 °C until future analysis. Eleven (*n* = 11) samples were excluded after quality control of the PBMCs. Low cell volume and poor cell viability were the reasons for exclusion, as they would compromise the validity of the assays performed. DNA damage response parameters were analyzed and assessed in HNSCC patients’ PBMCs at baseline. Fifteen (*n* = 15) healthy individuals (HC; 6 females/9 males; median age 57.9 years; range, 30–80) were also analyzed (protocol #BΠΠΚ, ΕΒΔ 509/10-07-2023). All participants provided informed consent in accordance with the Declaration of Helsinki, previously approved by the Ethics Committee of Attikon Hospital.

### 2.2. Cell Lines

Human immortalized normal skin fibroblasts, 1BR-3h-T cells, (kindly provided by Dr. Fousteri M., “Alexander Fleming”,Athens, Greece) were cultured in DMEM (Dulbecco’s modified Eagle’s medium), 10% FBS and 1% penicillin-streptomycin. Immortalized human B lymphocyte cells, RPMI-1788, were obtained from the American Type Culture Collection (ATCC), Manassas, VA, USA and cultured as recommended by the supplier. HNSCC cell lines UM-SCC-11A (laryngeal squamous cell carcinoma cells; provided by Thomas Carey University of Michigan, Ann Arbor), CAL-33 [tongue squamous cell carcinoma cells acquired from the Leibniz Institute DSMZ, Braunschweig, Germany; (ACC-447)] and BB49 (floor-of-mouth squamous cell carcinoma cells; kindly provided by Prof. Scorilas A., Department of Biology, NKUA, Athens, Greece), were expanded in DMEM, 10% FBS and 1% penicillin-streptomycin, 2mM glutamine, and 1% non-essential amino acids.

### 2.3. UVC Treatment

For ultraviolet irradiation, we utilized a Philips 6W germicidal lamp that primarily emits light in the UVC wavelength range. Both HNSCC and normal cell lines were irradiated with a total dose of 100 J/m^2^ and PBMCs were resuspended in PBS and irradiated with UVC, delivering a total dose of 5 J/m^2^. After the incubation in a complete medium at the appropriate time points, all cells were stored at −80 °C in a cryopreservation medium. The samples were subsequently analyzed to evaluate UVC-induced DNA damage burden levels.

### 2.4. Viability Assay

The cytotoxicity and cell proliferation analysis was performed using the sulforhodamine B (SRB) assay [26]. Briefly, 20,000 cells/well were seeded in 12-well plates, irradiated with 100 J/m^2,^ and incubated in a complete cell culture medium for 6 h. At each well, 1ml of 10% ice-cold trichloroacetic acid (TCA) was carefully added and the plates were incubated for 30 min on ice or overnight at 4 °C. After washing the plates and drying them, 500 μL of 0.4% SRB (Sigma–Aldrich, St. Louis, MO, USA) solution was added. The unbound SRB stain was washed with 1% acetic acid and the stained cells were dissolved with 500 μL of 10 mM Tris Base (pH 10.5). Absorbance was quantified using Tecan microplate reader, Männedorf, Switzerland, and cell viability was assessed compared to a non-irradiated negative control.

### 2.5. Alkaline Comet Assay

The alkaline single-cell gel electrophoresis assay was conducted following the previously described protocol [27]. Shortly suspended low-melting agarose cells were spread onto fully frosted pre-coated microscope slides. After exposure to lysis buffer at 4 °C for 1 h, electrophoresis was conducted for 30 min at 25 V and 255 mA. The slides were washed with neutralization buffer and ice-cold distilled H_2_O before drying. Gels were stained with SYBR Gold Nucleic Acid Gel Stain (Thermo Fischer Scientific, Waltham, MA, USA; # S11494) and analyzed using a fluorescence microscope (Zeiss Axiophot, Carl Zeiss, Jena, Germany). The Olive Tail Moment (OTM) parameter was calculated from at least 100 cells per treatment. Comet parameters were evaluated with the ImageJ/Open Comet software v1.3.1 (https://cometbio.org/).

### 2.6. Oxidative Stress and Apurinic/Apyrimidinic (Abasic; AP) Sites

Oxidative stress was quantified by a luminescence-based assay (Promega, Madison, WI, USA; #V6612), and AP sites were measured using an AP site assay kit (Cell Biolabs, San Diego, CA, USA; #STA-324). Both assays were conducted following the manufacturer’s protocol [28].

### 2.7. Statistical Analysis

We used unpaired *t*-test with Welch’s correction in order to compare continuous variables among the groups analyzed, or the non-parametric Mann–Whitney U test when normal distribution did not apply. Mean values ± standard deviations were used to present the results. GraphPad Prism 8.0.1 was used to perform statistical analysis. A *p*-value of <0.05 was considered statistically significant. The results were based on a minimum of three independent repeats.

## 3. Results

### 3.1. DNA Damage Repair and Oxidative Stress in HNSCC Cell Lines

**Figure 1:**
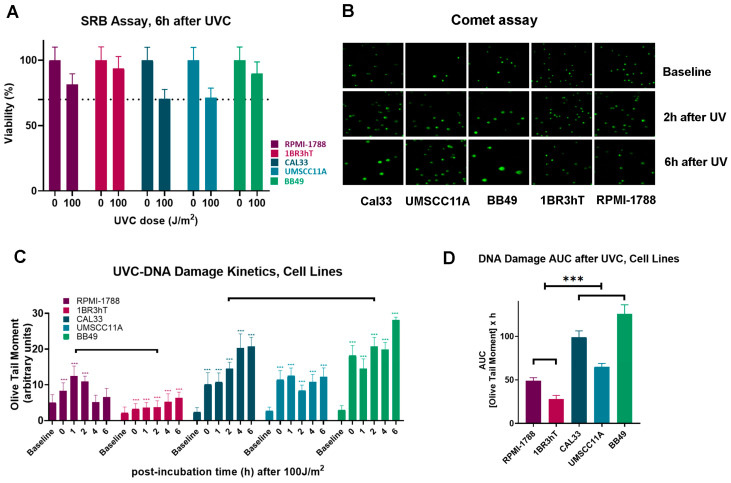
UVC-induced DNA damage repair in cell lines. (**A**) UVC-induced cytotoxicity and cell proliferation at 6 h. (**B**) Representative comet assay images of cell lines at baseline, 2 h and 6 h after UVC-treatment. (**C**) The UVC-induced DNA lesions kinetics measured by comet assay and (**D**) total amounts of DNA damage expressed as AUC. Error bars represent SD; *** *p* < 0.001.

The efficiency of DNA damage repair and the oxidative stress were studied in all three HNSCC cell lines (UM-SCC-11A, CAL33, BB49) and the two normal (RPMI-1788, 1BR3hT). First, using the SRB assay, UVC-induced cytotoxicity and cell proliferation were determined. We found that 6 h after UVC irradiation with a total dose of 100 J/m^2^ all cell types showed greater than 70% viability (Figure 1A). After cell irradiation with 100 J/m^2^ UVC and incubation for several time points (0, 1, 2, 4 and 6 h) in the appropriate culture media, the DNA damage was measured using comet assay that measures single-strand breaks (SSBs) and/or double-strand breaks (DSBs) (Figure 1B). Important differences in the DNA repair capacities were found between HNSCC and normal cell lines. When compared to normal cells, all HNSCC cell lines displayed lower DNA repair efficiencies (Figure 1C), leading to an increased UVC-induced DNA damage burden, expressed as the area under the curve (AUC) (*p* < 0.001; Figure 1D).

**Figure 2:**
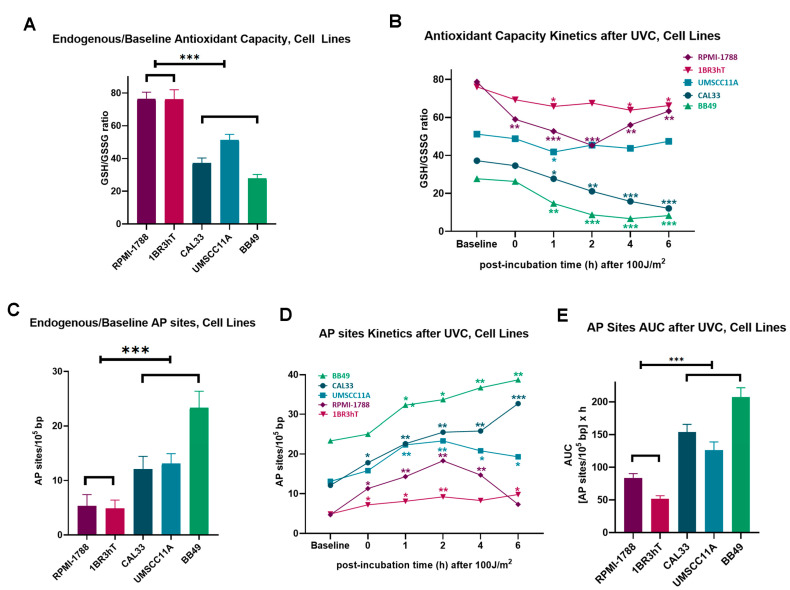
UVC-induced oxidative stress and AP sites in cell lines. Oxidative stress (**A**) at baseline and (**B**) 0, 1, 2, 4, 6h following UVC irradiation. AP sites (**C**) at baseline and (**D**) 0, 1, 2, 4, 6h following UVC irradiation. (**E**) Total amounts of AP sites expressed as AUC. Error bars represent SD; * *p* < 0.05, ** *p* < 0.01, *** *p* < 0.001.

Next, we evaluated the critical intracellular factors of oxidative stress and AP sites that are involved in cancer onset and progression, as well as in cancer therapy. All HNSCC cell lines showed significantly higher levels of endogenous/baseline and UVC-induced oxidative stress than normal cells, evidenced by the reduced GSH/GSSG ratio in HNSCC cells (*p* < 0.05; Figure 2A,B). In line with these data, higher levels of both endogenous/baseline and UVC-induced apurinic/apyrimidinic sites (all *p* < 0.05; Figure 2C,D) were observed in HNSCC cell lines when compared to normal cells, resulting in significantly increased accumulation of UVC-induced AP sites in malignant cells (*p* < 0.001; Figure 2E).

### 3.2. DNA Damage Repair and Oxidative Stress in PBMCs Derived from Patients with HNSCC

**Figure 3:**
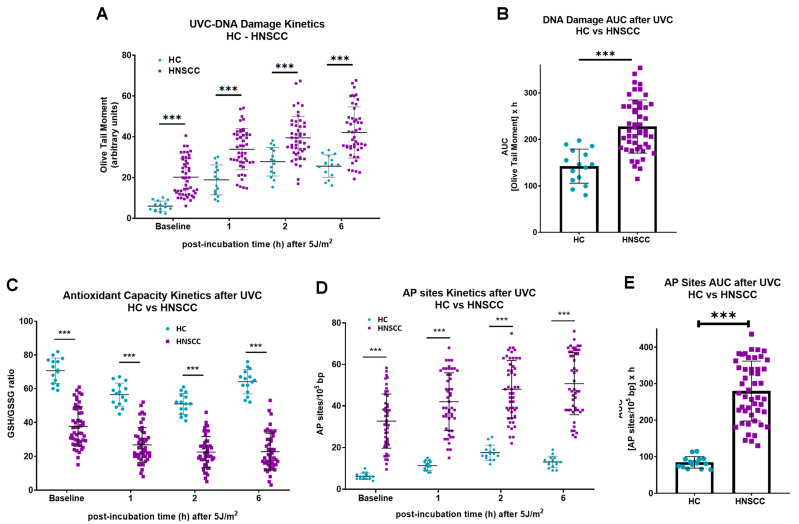
DNA damage repair, oxidative stress and AP sites in PBMCs from healthy controls and patients with HNSCC. (**A**) The kinetics of ultraviolet-induced DNA lesions and (**B**) total amounts of DNA adducts, expressed as AUC. (**C**) Oxidative stress and (**D**) AP sites kinetics in PBMCs from HC and HNSCC patients after ultraviolet irradiation. (**E**) Total amounts of AP sites after UVC irradiation expressed as AUC. Error bars represent SD; *** *p* < 0.001.

To ascertain whether the results obtained in cell lines can be extrapolated to samples from HNSCC patients, changes in the endogenous/baseline DNA damage, the DNA damage repair capacities, the oxidative stress, and the apurinic/apyrimidinic lesions were also analyzed in PBMCs from 15 healthy controls and 49 recurrent/metastatic HNSCC patients at baseline. Firstly, the endogenous/baseline DNA damage was measured using comet assay. We found that the levels of endogenous/baseline DNA damage were higher in PBMCs from HNSCC patients than those from healthy controls (*p* < 0.001; Figure 3A). In accordance with the results from the cell lines experiments, following irradiation of PBMCs with 5 J/m^2^, patients exhibited decreased DNA repair capacity compared to those from healthy controls (*p* < 0.001; Figure 3A), resulting in augmented accumulation of UVC-induced DNA damage in PBMCs from malignant patients (*p* < 0.001; Figure 3B). In addition, PBMCs from HNSCC patients exhibited higher endogenous/baseline and UVC-induced oxidative stress and AP sites than PBMCs from HC (Figure 3C,D), resulting in significantly increased accumulation of UVC-induced AP sites in samples from cancer patients (*p* < 0.001; Figure 3E).

**Figure 4:**
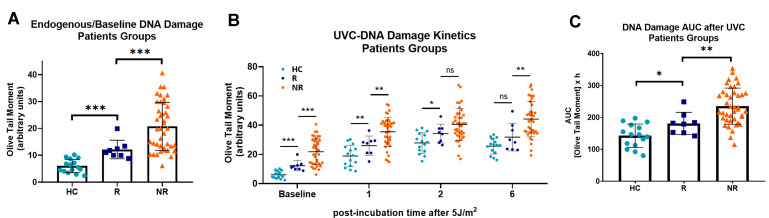
DNA damage repair in PBMCs from HNSCC patients and response to nivolumab therapy. (**A**) Endogenous/baseline DNA damage analyzed by comet assay in PBMCs from healthy controls (HC) and HNSCC patients groups differing in the response to nivolumab therapy. (**B**) The kinetics of UVC-induced DNA lesions and (**C**) total amounts of DNA adducts in healthy controls and HNSCC patients groups. Error bars represent SD; * *p* < 0.05, ** *p* < 0.01, *** *p* < 0.001, ns: not significant.

Next, following the stratification of HNSCC patients into responders (*n* = 8; CR and PR) and non-responders (*n* = 41; SD and PD), we tested the hypothesis that DNA damage-related parameters are involved in the response to nivolumab therapy. We found that the endogenous/baseline DNA damage was in the order HC < responders < non-responders, suggesting that non-responder HNSCC patients are characterized by higher levels of DNA damage than responders (all *p* < 0.001; Figure 4A). Moreover, the DNA repair capacity of PBMCs was analyzed after 5 J/m^2^ ultraviolet irradiation. Decreased DNA repair capacity was found in PBMCs derived from non-responders than PBMCs from responders (Figure 4B), leading to increased UVC-induced DNA damage burden in samples from non-responder patients (*p* < 0.05; Figure 4C).

**Figure 5:**
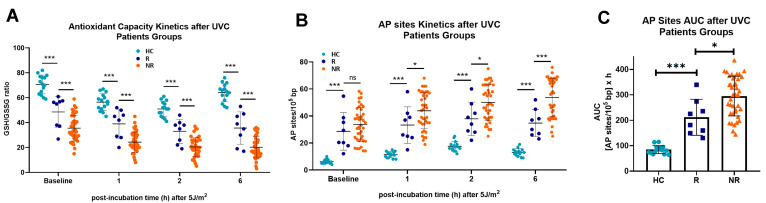
Oxidative stress and AP sites in PBMCs from patients and response to nivolumab therapy. (**A**) Oxidative stress and (**B**) AP sites kinetics following UVC irradiation in PBMCs from HC and patients differing in the response to nivolumab therapy. (**C**) Total amounts of AP sites after UVC irradiation. Error bars represent SD; * *p* < 0.05, *** *p* < 0.001, ns: not significant.

In addition, we found that PBMCs derived from non-responders to nivolumab therapy exhibited increased endogenous/baseline and UVC-induced oxidative stress and AP sites (Figure 5A,B), leading to increased accumulation of UVC-induced apurinic/apyrimidinic lesions in these patients (*p* < 0.05; Figure 5C).

Interestingly, when comparing CR/PR, SD, and PD separately, endogenous/baseline DNA damage increases, DNA repair capacity decreases, and oxidative stress/AP sites increases as the response progresses toward PD (Figures S2 and S3). However, most likely due to the small group size, many results did not reach statistical significance.

## 4. Discussion

Treatment with ICIs is a rapidly evolving approach and the gold standard treatment for recurrent and metastatic HNSCC [29,30]. Although nivolumab and pembrolizumab approval represents a significant advancement in the oncology field, only a small proportion of patients derive benefits from blocking the PD-1/PD-L1 pathway, raising the need for predictive biomarkers and the development of combination strategies to augment therapeutic efficacy [30]. Interestingly, recent reports have demonstrated that the DDR network interacts with the immune system, regulating host immunological responses and potentially offering a novel tool for improving immunotherapy efficacy [16]. Hence, herein we studied DDR-associated signals and oxidative stress in HNSCC patients with different responses to immune checkpoint blockade.

Since DNA damage can result in mutagenesis, genomic instability, and cell death, it poses a serious danger to cell survival [31]. In the present study, higher levels of endogenous/baseline DNA adducts were observed in HNSCC patients than in healthy controls, with non-responders to nivolumab therapy presenting the highest values. To gain a clearer insight into the cause of this phenomenon, we first examined the DNA repair efficiency in PBMCs from patients with HNSCC. We observed that these patients showed defective DNA repair capacity compared with HC, with non-responders exhibiting the lowest DNA repair capacity. These findings were consistent with earlier results indicating that critical DNA repair pathways, namely nucleotide excision repair (NER), DSBs repair, base-excision repair (BER), and mismatch repair (MMR) are defective in HNSCC cells. Indeed, previous reports have shown decreased NER capacity in cancer patients compared with healthy controls, with responders to chemotherapy exhibiting decreased NER capacity than non-responders [13,32,33,34,35]. To explain the lower NER efficiency of patients with HNSCC, previous studies showed decreased expression of important NER genes in these patients than healthy controls [36]. Another research pointed out that polymorphisms in several NER genes are linked to the development and course of HNSCC, as well as the response to treatment [37]. Furthermore, previous studies have also found that the DSB repair mechanism is also deregulated in HNSCC patients. Shammas and colleagues [38] found that a permanently upregulated DSB repair pathway may promote the development and spread of tumors and the emergence of a phenotype of drug-resistance. In line with these findings, we previously showed that HNSCC patients had a higher DSB repair capacity than healthy controls and that important genes linked to DSB repair showed increased expression in HNSCC patients [13]. Moreover, according to earlier investigations, several DSB repair genes polymorphisms are involved in an increased risk of HNSCC development [39,40]. Furthermore, other studies have shown that the expression of some base excision repair genes was lower in patients with HNSCC patients than in healthy controls, and a number of MMR genes were overexpressed. Other studies also reported that polymorphisms in genes related to MMR and BER might play a role in the progression of the disease [41,42].

In addition, we assessed the induction of oxidative stress and apurinic/apyrimidinic lesions in order to determine the cause of the elevated production of DNA damage in HNSCC patients. In accordance with our previous study [13], we observed that in comparison to healthy controls, HNSCC patients had higher levels of both these factors. It is recognized that oxidative stress plays a pivotal role in the onset and progression of HNSCC. Indeed, prior research has shown a strong association between head and neck cancer and oxidative stress, as alcohol consumption and tobacco use (both known to increase the production of ROS) are recognized as critical etiological factors for this cancer [13,43]. Intracellular ROS cause oxidative DNA damage, which leads to single- and double-strand breaks, apurinic/apyrimidinic lesions, and changes in DNA bases, with many of these lesions being hazardous and/or mutagenic [44]. For example, elevated levels of mutagenic 8-hydroxyguanine lesions are found in cancer and aging cells [45]. Moreover, it has been demonstrated that microsatellite instability, which is found most often in colorectal, endometrial, and gastric cancer, may also be caused by H_2_O_2_-induced oxidative DNA damage [46]. Importantly, herein we found significant differences in the oxidative stress status between HNSCC patients with different responses to nivolumab treatment, with responders showing significantly lower oxidative stress compared with non-responders. These results are in accordance with our previous study, which showed that lower oxidative stress correlates with longer PFS of HNSCC patients after platinum-based therapy [13]. Other studies have also reported that patients with HNSCC with decreased oxidative stress have a reduced risk of tumor recurrence after concomitant chemoradiotherapy, indicating that that tumors with higher oxidative stress exhibit more aggressive behavior [47]. Moreover, apurinic/apyrimidinic sites are extremely cytotoxic and mutagenic DNA lesions that are formed in the genome from a variety of endogenous and exogenous causes. Interestingly, over 10^4^ apurinic/apyrimidinic lesions are spontaneously formed in the human genome per day, and this load can be significantly increased by exposure to exogenous damaging substances [48]. When DNA repair enzymes cleave apurinic/apyrimidinic sites, DNA SSBs with blocked ends are formed. These single-strand breaks are not substrates for DNA ligases or DNA polymerases [49] and can be transformed into extremely cytotoxic double-strand breaks following DNA replication [50]. Since ROS cause apurinic/apyrimidinic sites [51], the increased AP sites levels observed in the HNSCC patients studied here may be partly explained by the elevated oxidative stress of HNSCC patients. Notably, the cell line experiments presented above showed corresponding results on oxidative stress and DNA damage repair-associated signals, confirming the wide application of our findings.

Together, significant differences in the oxidative stress levels and the DNA damage repair capacity were observed between responders and non-responders to nivolumab therapy. These results showed a strong correlation between deregulated DDR-related parameters in peripheral blood mononuclear cells derived from patients with HNSCC at baseline and the response to subsequent immune checkpoint blockade therapy. Indeed, extensive observations suggested that the therapeutic effect of the ICIs is directly impacted by the tumors’ altered DDR pathway, which influences immunogenicity and immune cell infiltration [52,53,54,55]. Currently, the tumor mutational burden [56,57], MMR deficiency [58], and PD-L1 expression levels [59] are the main indicators used to predict the impact of ICI therapy on cancer patients. Prior research on solid tumors and hematological malignancies has demonstrated that blood DDR parameters monitoring may help predict patient’s prognosis and forecast the response to chemotherapy treatment [13,23,32,34,35,37,41,47,55]. Therefore, the results presented here open the prospect of evaluating the effectiveness of immune checkpoint blockade by measuring oxidative stress and DNA damage repair status in a readily accessible tissue such as PBMCs.

Our study has certain limitations. First, all patients received nivolumab as a second-line treatment, which followed either first-line chemotherapy with the EXTREME regimen (platinum/5-FU/cetuximab) or disease recurrence occurring within six months of completing cisplatin-based chemoradiotherapy for locally advanced disease. Although peripheral blood samples were collected from patients just before the start of immunotherapy, conducting correlation analyses related to prior treatment lines is challenging due to the small sample size. Moreover, while PD-L1 is a well-known predictive biomarker for immunotherapy that could have affected our results, the limitations of the data and the small sample size complicate our ability to determine whether the response is more closely related to PD-L1 expression rather than DNA damage burden, repair capacity, or a combination of these factors. Finally, we did not examine the mechanistic link between DDR-associated parameters (DNA damage repair mechanisms, signaling pathways, cell cycle, apoptosis, etc.) and the response to immune checkpoint blockade. These are issues to be addressed in our future research.

## 5. Conclusions

Two important mechanisms for the survival of living organisms are the immune system and the DDR network. Both these mechanisms play important roles in the onset and progression of a number of diseases as well as in the response to chemotherapy. According to recent data, these two systems work together to support the coordinated operation of multicellular organisms. Thus, herein we examined the connection between the anticancer efficacy of ICI-based treatment and the DDR-related parameters measured in peripheral blood mononuclear cells derived from HNSCC patients. Compared to non-responders, we observed that nivolumab therapy responders had lower endogenous/baseline DNA damage, higher DNA repair capacities, less oxidative stress, and decreased apurinic/apyrimidinic lesions. These results, when properly validated, can lead to the discovery of new predictive biomarkers to immunotherapies, and the design of novel combination regimens, including DDR-targeted drugs and immune checkpoint inhibitors.

## Figures and Tables

**Table 1:** Patients’ clinicopathological characteristics.

Characteristic	Patient Cohort *n* (%)		
Age (Years) Median (min, max)	65 (48, 93)		
Sex	Male	40	(81, 6)
	Female	9	(18, 4)
Smoking	Non smoker	9	(18, 4)
	Light smoker	6	(12, 2)
	Heavy smoker	27	(55, 1)
	N/A	7	(14, 3)
Alcohol	No/Social	23	(46, 9)
	Light	1	(2, 0)
	Heavy	17	(34, 7)
	N/A	8	(16, 4)
Primary site	Lip/Oral cavity	18	(36, 7)
	Oropharynx	13	(26, 5)
	Larynx	16	(32, 7)
	Other	2	(4, 1)
HPV status (oropharynx)	Positive	2	(15, 3)
	Negative	8	(61, 5)
	N/A	3	(23, 1)
Stage	Metastatic	17	(34, 7)
	Recurrent	32	(65, 3)
PD-L1 CPS baseline	<1	16	(32, 7)
	1–19	9	(18, 4)
	≥20	8	(16, 3)
	N/A	16	(32, 6)
Best response to immunotherapy	CR/PR	8	(16, 3)
	SD	6	(12, 2)
	PD	35	(71, 5)

N/A, not applicable; CR, complete response; PR, partial response; SD, stable disease; PD, progressive disease.

## Data Availability

The data presented in this study are available by specific request to the corresponding author.

## Supplementary Materials

The following supporting information can be downloaded at: www.mdpi.com/article/10.3390/biology14020195/s1, Figure S1. CONSORT diagram; Figure S2. DNA damage repair in PBMCs from HNSCC patients and response to nivolumab therapy; Figure S3. Oxidative stress and AP sites in PBMCs from HNSCC patients and response to nivolumab therapy.
