# Supplementary material for: UVC-Induced Oxidative Stress and DNA Damage Repair Status in Head and Neck Squamous Cell Carcinoma Patients with Different Responses to Nivolumab Therapy
Source: Biology (Basel). 2025 Feb 13;14(2):195. doi: 10.3390/biology14020195 (PMC11852043; doi:10.3390/biology14020195)
Supplement: Supplementary file 1 [file biology-14-00195-s001.zip › biology-3464253-supplementary.pdf]

Supplementary Data

UVC-Induced Oxidative Stress and DNA Damage Repair Status in Head and Neck Squamous Cell Carcinoma Patients with Different Responses to Nivolumab Therapy

Christina Papanikolaou 1, Panagiota Economopoulou 2, Niki Gavrielatou 2, Dimitra Mavroeidi 1, Amanda Psyrri 2 and Vassilis L. Souliotis 1,*

1 Institute of Chemical Biology, National Hellenic Research Foundation, 11635 Athens, Greece

2 Second Department of Internal Medicine, Medical Oncology Section, National and Kapodistrian University of Athens, Attikon University Hospital, 12462 Athens, Greece
* Correspondence: vls@eie.gr

Table of Contents

- Figure S1. CONSORT diagram
- Figure S2. DNA damage repair in PBMCs from HNSCC patients and response to nivolumab therapy
- Figure S3. Oxidative stress and AP-sites in PBMCs from HNSCC patients and response to nivolumab therapy

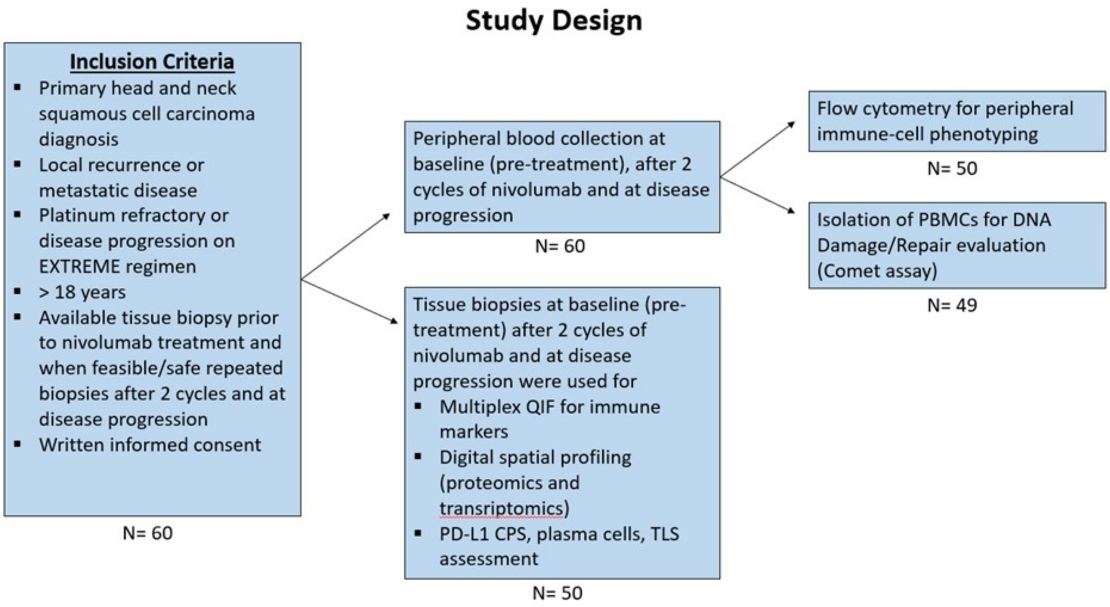

Figure S1. CONSORT diagram

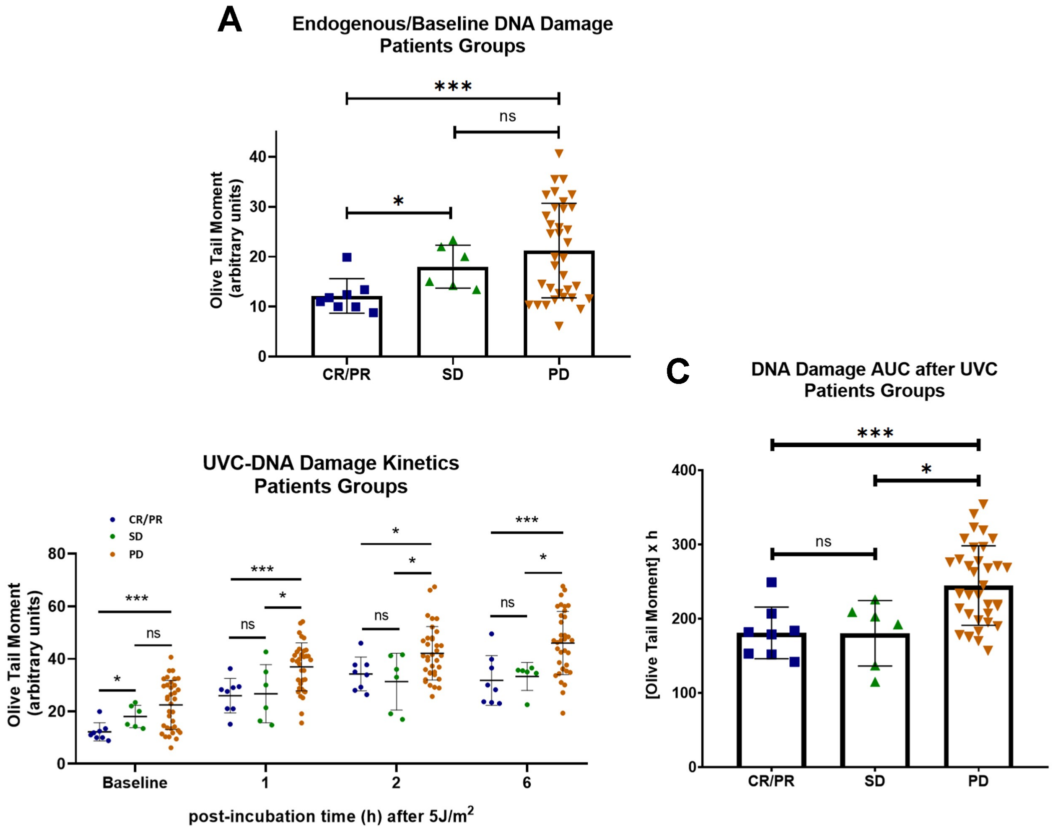

Figure S2. DNA damage repair in PBMCs from HNSCC patients and response to nivolumab therapy. (A) Endogenous/baseline DNA damage measured by alkaline comet assay in PBMCs from HNSCC patient groups differing in the response to nivolumab therapy. (B) The kinetics of UVC-induced DNA lesions and (C) total amounts of DNA lesions, expressed as AUC, in HNSCC patient response groups. Error bars represent SD; *P < 0.05, ***P < 0.001, ns: not significant. The results were based on a minimum of three independent repeats.

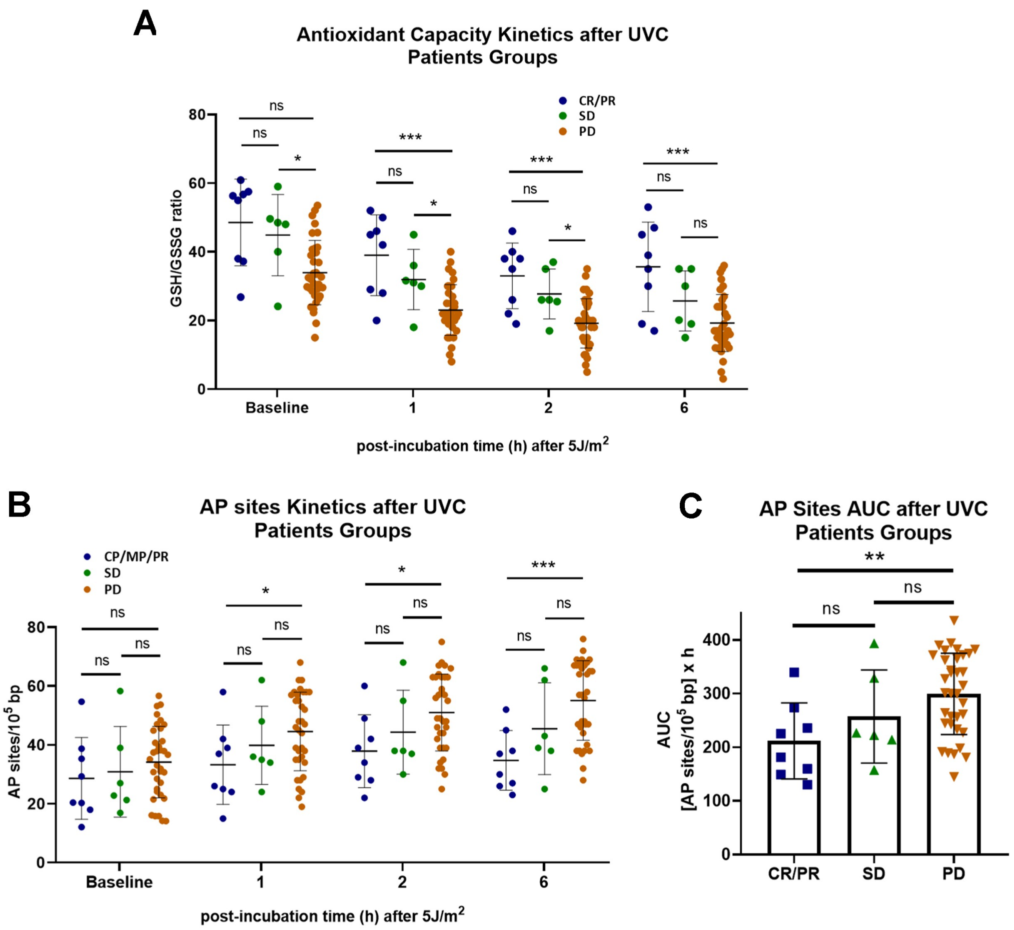

Figure S3. Oxidative stress and AP-sites in PBMCs from HNSCC patients and response to nivolumab therapy. (A) Oxidative stress and (B) AP-sites kinetics following UVC irradiation in PBMCs from HNSCC patients differing in the response to nivolumab therapy. (C) Total amounts of AP-sites, expressed as AUC, after UVC irradiation. Error bars represent SD; **P* < 0.05, **P < 0.01, ***P < 0.001, ns: not significant. The results were based on a minimum of three independent repeats.
